# A case of tubulointerstitial nephritis in a patient with an influenza H1N1 infection

**DOI:** 10.1007/s00467-012-2201-1

**Published:** 2012-06-17

**Authors:** Niloufar Ashtiani, Margot F. Mulder, Joanna A. E. van Wijk, Arend Bokenkamp

**Affiliations:** 1 Department of Pediatrics, VU University Medical Center, De Boelelaan 1117, 1081 HV Amsterdam, The Netherlands; 2 Department of Metabolic Disorders, VU University Medical Center, Amsterdam, the Netherlands; 3 Department of Pediatric Nephrology, VU University Medical Center, Amsterdam, the Netherlands

## Abstract

**Background:**

Patients suffering from an H1N1 infection mainly suffer from respiratory symptoms but may also develop symptoms in other organ systems, such as the kidneys.

**Case-diagnosis/treatment:**

A 4 ½ year-old boy was admitted with relatively mild respiratory symptoms of H1N1 infection, but developed severe generalized proximal tubular dysfunction with sterile leucocyturia and a reversible rise in serum creatinine. He made a full recovery with supportive therapy.

**Conclusion:**

Influenza H1N1 may be associated with acute tubulointerstitial nephritis.

## Introduction

An H1N1 (influenza A) pandemic emerged in 2009 resulting in a significant burden of illness. These patients suffered mainly from respiratory symptoms, but some also developed symptoms in other organ systems. Acute kidney injury appeared common in critically ill adult patients with H1N1 infection. A recent study revealed an incidence of 34 % of acute kidney injury among 628 ICU patients with H1N1 infection [1]. The data available for pediatric patients mostly relates to patients with underlying kidney disease and describes a wide range in incidence [2]. There are two case reports on children with H1N1 infection who developed acute glomerulonephritis [3, 4].

Here, we present the case of a child with tubulointerstitial nephritis during H1N1 infection.

## Case report

A 4½ year-old boy with arthrogryposis multiplex congenita of unknown origin developed fever, cough and vomiting during 3 days before presenting at the emergency room. Besides paracetamol (60 mg/kg/day), he had not taken any medication. Physical examination revealed a heart rate of 90 beats per minute, mild signs of dehydration with a weight loss of 1 kg in the preceding days, while urine output was maintained. The lungs were clear on auscultation, a chest X-ray showed no significant abnormalities. He was admitted under the diagnosis of a viral infection and received supplemental oxygen. Polymerase Chain Reaction in a nasal swab was positive for influenza H1N1. Oseltamivir was withheld because symptoms had started more than 48 hours before presentation. Paracetamol was continued during admission.

Laboratory results on admission showed a severe hypokalemia and hypophosphatemia in combination with mild hyponatremia due to increased renal losses. In addition, he had ketoacidosis, plasma lactate was normal. Serum creatinine had risen from a baseline of 11 μmol/l at a recent outpatient visit to 19 μmol/l, corresponding to acute kidney injury pRIFLE - “R” [5]. Urine analysis was remarkable for sterile leucocyturia and low molecular weight proteinuria. Renal ultrasound was normal.

He was started on enteral feeding via a nasogastric tube and received enteral and parenteral supplementation of sodium, potassium, magnesium and phosphate. During the following days the patient developed an incomplete renal Fanconi syndrome (Table 1) with low molecular weight proteinuria and loss of sodium, potassium, phosphate, magnesium and uric acid and a de novo generalized hyperaminoaciduria. Renal (ie normal anion-gap) acidosis and glucosuria were absent.

**Table 1:** Laboratory findings during the course of the disease

Laboratory results	Day 1	Day 13	Day 45	Reference	Conversion factor to metric units
Blood					
Creatinine (μmol/l)	19	11	12	n.a.	mg/dl × 0.011
Urea (mmol/l)	5.5	4.7	3.6	3.0-7.5	mg/dl × 6.006
Uric acid (μmol/l)	552	65	225	200–450	mg/dl × 0.017
Na (mmol/l)	135	142	139	136–146	mg/dl × 2,298
K (mmol/l)	1.6	4.4	4.2	3.6-4.8	mg/dl × 3.910
Cl (mmol/l)	92	106	104	98–108	mg/dl × 3.545
Mg (mmol/l)	0.61	0.86	0.84	0.70-1.00	mg/dl × 2.431
Ca (mmol/l)	1.92	2.32	2.51	2.20-2.60	mg/dl × 4.008
P (mmol/l)	0.40	1.69	1.82	0.70-1.40	mg/dl × 3.097
pH	7.33	7.40	7.40	7.35-7.45	n.a.
pCO_2_ (mmHg)	24	36	37	35–45	n.a.
HCO_3_ (mmol/l)	12.1	21.8	22.3	22–26	n.a.
Urine					
Ketostix	3+	n.d.	n.d.		
Fractional Excretion urea (%)	18.0	n.d.	n.d.		
Fractional Excretion uric acid (%)	11.9	57.4	11.2		
Fractional Excretion Na (%)	2.58	1.87	1.01		
Fractional Excretion K (%)	112	23.6	8.96		
Fractional Excretion Mg (%)	11.4	12.1	5.64		
Tubular maximum Phosphate					
reabsorption /Glomerular Filtation Rate	-0.82	1.20	1.64		
α-1 microglobuline (mg/l)	11.8	n.d.	< 4.0		
β-2 microglobuline (μg/l)	6330	118	48		
Albumine/Creatinine ratio (mg/mmol)	32	5	9		
α-1 microglobuline/Creat (mg/mmol)	10.2	n.d.	< 2		
β-2 microglobuline/Creat (μg/mmol)	5466	59.8	26.0		
Aminoaciduria	3+, gen	1+, gen	1+, select		

Legend:

gen, generalized; select, selective; n.a., not applicable; n.d., not done

Serum creatinine, as well as tubular function, improved within one week and had normalized after six weeks and have been normal since.

Extensive metabolic testing during evaluation for arthrogryposis multiplex as well as during the current illness revealed no signs of an underlying metabolic defect.

## Discussion

Here we present a patient with acute tubulointerstitial nephritis concomitant with an influenza H1N1 infection. The diagnosis is based on the combination of an incomplete renal Fanconi syndrome, non-oliguric renal failure and the presence of sterile leucocyturia. In view of the rapid improvement of renal dysfunction and the respiratory symptoms we refrained from performing a kidney biopsy to confirm our clinical diagnosis.

The differential diagnosis of acute proximal tubular dysfunction in our patient includes the manifestation of an underlying mitochondrial disease, which might also have caused the arthrogryposis. Yet, extensive metabolic testing in blood and urine yielded no signs of mitochondriopathy. History revealed the use of paracetamol, which has been associated with acute tubulointerstital nephritis in two patients with a concomitant alcohol intoxication [6, 7]. Rapid reversibility and the absence of any systemic signs of autoimmunity render an underlying immune disorder (eg systemic lupus erythematosus, sarcoidosis) unlikely. Since many viruses have been linked with tubulointerstitial nephritis, we postulate a causal relationship between the proven influenza H1N1 infection. Still, we did not rule out other infections known to cause acute tubulointerstitial nephritis such as Cytomegalovirus, Epstein-Barr virus, Hepatitis C virus, human immunodeficiency virus, mycoplasma, or Hanta virus.

Several pathological studies have demonstrated that H1N1 virus is not restricted to the lungs. In one study H1N1 virus was found in the cytoplasm of glomerular macrophages [8]. Nin et al demonstrated the virus in epithelial cells in the capsule of Bowman and in distal tubular cells [9].

Most patients with H1N1-related renal injury suffer from acute tubular necrosis during intensive care therapy. Nin et al distinguish early versus late kidney injury during H1N1 infection. The former is reversible reflecting hemodynamic instability, whereas in the latter persistent injury and/or co-morbidity might play a role [10]. Therefore, this group bears a worse prognosis and these patients often need renal replacement therapy.

To the best of our knowledge, tubulointerstitial nephritis has not been reported previously in patients with influenza H1N1 infection. This may reflect under-reporting as the signs of tubulointerstial nephritis can be relatively mild, and therefore the diagnosis be missed unless blood and urine are tested.

In conclusion, we describe a 4½ year-old boy with acute tubulointerstitial nephritis during an influenza H1N1 infection, with full recovery of renal function after supportive treatment.
